# Isomalto-oligosaccharides ameliorate visceral hyperalgesia with repair damage of ileal epithelial ultrastructure in rats

**DOI:** 10.1371/journal.pone.0175276

**Published:** 2017-04-24

**Authors:** Weida Wang, Haiwei Xin, Xiucai Fang, Hongtao Dou, Fangyi Liu, Dan Huang, Shaomei Han, Guijun Fei, Liming Zhu, Shenghua Zha, Hong Zhang, Meiyun Ke

**Affiliations:** 1 Department of Gastroenterology, Peking Union Medical College Hospital, Chinese Academy of Medical Sciences and Peking Union Medical College, Beijing, China; 2 Department of Clinical Laboratory, Peking Union Medical College Hospital, Chinese Academy of Medical Sciences and Peking Union Medical College, Beijing, China; 3 Department of Gastroenterology, The People’s Hospital of Guangxi Zhuang Autonomous Region, Nanning, China; 4 Department of Epidemiology and Statistics, Institute of Basic Medical Sciences, Chinese Academy of Medical Sciences and School of Basic Medicine, Peking Union Medical College, Beijing, China; 5 Beijing Tongrentang Health-Pharmaceutical Co., Ltd., Beijing, China; Center for Cancer Research, UNITED STATES

## Abstract

**Background:**

Treatment of irritable bowel syndrome (IBS) with probiotics has achieved effectiveness to a certain extent. Whether prebiotics will work is still unclear. This study aimed to investigate the therapeutic effects of the prebiotic isomalto-oligosaccharides (IMO) on visceral hypersensitivity (VHS) in rats and to explore potential mechanism.

**Methods:**

Water avoidance stress (WAS) was used to induce VHS in rats. The score for the abdominal withdrawal reflex (AWR) was determined while colorectal distension and compared between VHS group and control group in order to validate VHS preparation. Rats with VHS were then divided into an IMO-treated group (intragastric 5% IMO, 2 mL/d, 14 days) and a water-control group (intragastric water). After treatment, AWR score and intestinal transit rate (ITR) were determined, stool culture was performed, the ultrastructure of the ileum epithelium was observed with scanning electron microscopy (SEM), and serum cytokines were measured.

**Results:**

WAS significantly increased AWR score responding to colorectal distension, and lowered the pain threshold. IMO treatment improved VHS with a reduction in AWR score on graded colorectal distension and an increase in pain threshold. SEM showed damages on the ileal epithelial ultrastructure in VHS rats, which was attenuated by IMO treatment. ITR, fecal microbiota and serum cytokine levels were comparable among control group, water-control group, and IMO-treated rats.

**Conclusion:**

In this randomized placebo-controlled study, the results showed that IMO ameliorated WAS-induced visceral hyperalgesia in rats, this effect may be attributed to the repair of damages on intestinal epithelial ultrastructure.

## Introduction

Irritable bowel syndrome (IBS) is a physiological-psychological-social disease, and its etiology is related to genetics, foods, infection, and mental factors. The pathogenesis of IBS has been ascribed to visceral hypersensitivity (VHS), gastrointestinal dysmotility, and dysfunction of the brain-gut axis. Treatment approaches in IBS are mainly focused on symptoms management through lifestyle modification, psychotherapy and pharmacotherapy. These conventional pharmacological treatments include antispasmodics, antidiarrheals, or laxatives and bulking agents, 5-hydroxytryptamine-3 (5-HT3) receptor antagonists, 5-HT4 receptor agonists, antibiotics, tricyclic antidepressants, selective serotonin reuptake inhibitors, and probiotics. Among these, medications that alleviate visceral hyperalgesia were insufficent. Several randomized controlled trials and systemic reviews indicated that probiotics administration was to some extent better than placebo in improving overall symptoms such as pain, flatulence and bloating[1–6], the effects that may be associated with the inhibition of gut flora adhesion and translocation[7,8], regulation of the localized intestinal immune response[9,10], maintenance of intestinal mucosal permeability, modulation of nociceptive bacterial metabolites production, and thereby decrease the threshold of visceral pain[11–14].

Prebiotics belong to a group of dietary supplements that are non-digestible, but can stimulate the growth and/or activity of bacteria in the digestive system in ways beneficial to health. Experimental data and human studies have shown beneficial effects of prebiotic supplementation in different pathological conditions, including some gastrointestinal diseases such as liver diseases, IBD and chronic constipation[15–17]. However, there is no consensus on the therapeutic efficacy of prebiotics in IBS[18]. The non-fermentable oligosaccharides, isomalto-oligosaccharides(IMO), are produced from starch by enzymatic conversion, having α(1,6) and α(1,4) glycosidic bonds. As a food additive, IMO have some merits such as low calories, low sweetness and non-toxicity. In physiological studies in animals, IMO have been shown to promote the growth of *Lactobacillus* and *Bifidobacterium*. Its metabolite short-chain fatty acid(SCFA) can reduce intestinal pH and provide energy for colonic epithelial cells. And the increased *Lactobacillus* number may improve local and systemic Th-1 like immune response and regulation of immune function[19,20]. Similar findings have been observed in clinical trials[21]. Although therapeutic efficacy of IMO has not been reported in non-constipation predominant IBS, we speculated that IMO might exert a therapeutic effect on IBS to a certain extent, because IBS patients have a high incidence of abnormal gut microbiota[22,23].

The chronic stress model, water avoidance stress (WAS), can induce anxiety-like behaviors and visceral hyperalgesia accompanied by increases in pro-inflammatory cytokines (IL-1β and IFN-γ) and numbers of mucosal mast cells. Thus, WAS may be used to induce the clinical manifestations of visceral hyperalgesia due to IBS and has been used to establish an IBS animal model[24]. The current study aimed to investigate the therapeutic efficacy of IMO in IBS rats and explore potential mechanisms. Our findings have been presented in the Joint International Neurogastroenterology and Motility Meeting (Neurogastroenterology and Motility, 2012, 24, Supplement 2).

## Materials and methods

### Animals

Male Wistar rats (specific pathogen free, SPF) weighing 160–180 g were purchased from the Experimental Animal Center of Chinese Military Academy of Medical Sciences. Experimental animals were bred in the Experimental Animal Center of Chinese Military Academy of Medical Sciences until about 100days. After that, animals were housed in the Experimental Animal Laboratory of Peking Union Medical Collage Hospital in a SPF environment (n = 3 per cage). The animal administration was centralized by the Experimental Animal Laboratory according uniform protocol. Ventilation was performed in the presence of a filter for each cage in a closed environment with a 12h/12h light/dark cycle. Room temperature was controlled at 20–24°C and humidity was 50%. Animals were given *ad libitum* access to food and water, and allowed to accommodate to the environment for 3 days before experimental use. Investigators visited animals twice a day to exchange water and feed supply. Daily weight and stool trait were measured and recorded every day to monitor animal health. Vitality signs were also observed. Animals recieved inhalation anesthesia before invasive procedures and were sacrificed by neck dislocation at the end of experiment time. None of animals died because of severely ill. Two animals were died because of technical hitch of air vent of a rearing cage. The cause of death was suffocation. Four animals died during AWR measurement because of anesthetic accident. Our intervention was minimal invasive and we assumed that there would be no animals dying from intervention complications. But if any of the animals were severely ill they were not suitable for evaluation or further experiment. Euthanasia procedure would be performed. Vital signs would be major clinical signs to detemine whether or when to euthanasia. This study was approved by the Ethics Committee of Peking Union Medical Collage Hospital.

### Water avoidance stress induction

WAS was introduced as previously reported[24]. On the basis of findings in a pilot study, the length, width and height of the white translucent water tank used to induce WAS were set to 64 cm, 40 cm and 35 cm, respectively. At the center of this tank, a transparent glass platform (8 cm × 8 cm × 15 cm) was placed and water (temperature 25°C) was added to the tank to a level 1 cm below the platform. Rats were randomly assigned to control group or WAS group in a proportional distribution of 1:2. In the morning, rats in the WAS group were placed on the platform and allowed to stay on it for 1 h. This protocol was repeated for 10 days. In the control group, rats were placed in another identical tank for 1 h at the same time, but without water. After modeling, this WAS group was randomized into water-control group (WAS and water gavage) and IMO-treated group (WAS and IMO gavage). The number of fecal particles and the number of unformed fecal particles (Bristol Stool Scale 5–7) in the tank excreted during the hour were counted.

### Treatment

IMO was provided by Beijing Tongrentang Health-Pharmaceutical Co., Ltd. China. To prepare the 5% IMO solution, 5 g of IMO powder were dissolved in 100 mL of distilled water and sterilized under ultraviolet radiation for 30 min. The IMO solution was then stored at room temperature for use. As previously mentioned, rats in the WAS group were randomly assigned into IMO-treated group and water-control group. In the IMO-treated group, rats were given 2 mL of 5% IMO solution by gastric lavage twice daily for the following 14 days. In the water-control group, rats were given 2 mL distilled water by gastric lavage twice daily for 14 days. In control group, rats received no gavage at the same period. There were 9 rats in each group.

### Evaluation of visceral hypersensitivity

VHS was evaluated as previously reported[25]. A latex balloon (4 cm in length) was connected to a pressure measuring device (with constant pressure). At 8–11 AM, abdominal withdrawal reflex (AWR) was measured. Rats were fasted for 18 h before this measurement was performed. Following anesthesia with sevoflurane, a paraffin oil coated balloon was inserted into the rectum with the tail of the balloon 1 cm from the anus. The tube attached to the balloon was fixed, and the animals were placed in a transparent box for observation. Animals were allowed to accommodate to the environment for 30 min. The balloon was then distended with distention pressures of 20, 40, 60 and 80 mmHg. Distention was done rapidly (within 3 s) and sustained for 30 s with an interval of 4 min between distentions. Distention was performed three times at each pressure, and the corresponding AWR scores were recorded. Threshold in these measurements refers to the minimal distension pressure that caused the rat’s lower abdominal wall to move away from the platform or induced the flattening of the abdominal wall (AWR score = 3). AWR was evaluated by a specific investigator who was blinded to the study assignments. VHS was measured twice (one measurement was done after establishment of the WAS model and the other after treatment with IMO or water). In the present study, experimental animals were performed with three batches of rats, AWR scores were only determined in the first two batches.

### Detection of intestinal transit rate

At the end of the treatment period, animals were fasted for 24 h, and then received intragastric lavage with 10% activated carbon solution (2 mL). Forty-five minutes later, they were sacrificed by cervical dislocation. Laparotomy was performed immediately, and the whole bowel taken out of the abdominal cavity and moistened with PBS. The bowel was placed on a table (but not stretched) and the length of the whole bowel and length of bowel containing the activated carbon were measured independently. The intestinal transit rate (ITR) was calculated as follows: ITR = [length of the activated carbon moving in the bowel (cm) / length of the whole bowel (cm)] ×100%.

### Isolation and culture of gut microbiota

Vancomycin and kanamycin used for preparation of KVLB medium were purchased from Sigma (USA). Fresh stool was collected by abdominal compression, and smeared on different media after dilution (Brandt anaerobic medium for *Escherichia coli*; Brandt anaerobic medium for *Lactobacillus*; KVLB medium for *Bacteroides*). Anaerobic culture was done in a bag for 72 h, and the number of viable bacterial colonies was determined. The logarithm of colony number to the base 10 was calculated as follows: log10 (CFU g^-1^) = [sample weight (g) + volume of diluted solution (ml)] / sample weight (g) × folds of dilution × number of viable bacterial colonies.

### Detection of ultrastructure of ileum mucosa by scanning electron microscopy

After treatment, 2 rats were randomly selected from each group and sacrificed. The ileum was collected at 5 cm from the ileocecal site and opened along the mesentery. The ileum was then flushed with cold normal saline and rapidly cut into blocks (3–5 mm^2^), followed by fixing in cold 4% glutaraldehyde solution and storage at 4°C. Scanning electron microscopy (SEM, Hitach, S-3500N) was used to observe the ultrastructure of the intestinal mucosa.

### Detection of serum cytokines by ELISA

Serum cytokines (IL-10, IL-12, and TNF-α) concentrations were assessed by using enzyme-linked immunosorbent assay (ELISA) kits (Genetimes Technology, Inc).

## Statistical analysis

Data were expressed as median and interquartile (IQR). One way analysis of variance, independent sample *t* test, repeated measures *t* test and Wilcoxon sum rank test were used for comparisons of numeric data. Least significance difference (LSD) test was used to conduct multiple comparisons of numeric data. Statistical analysis was performed with SPSS version 18.0. A value of *P* < 0.05 was considered statistically significant.

## Results

### Water avoidance stress mimics the manifestations of IBS-D: Diarrhea and visceral hypersensitivity

**Fig 1:**
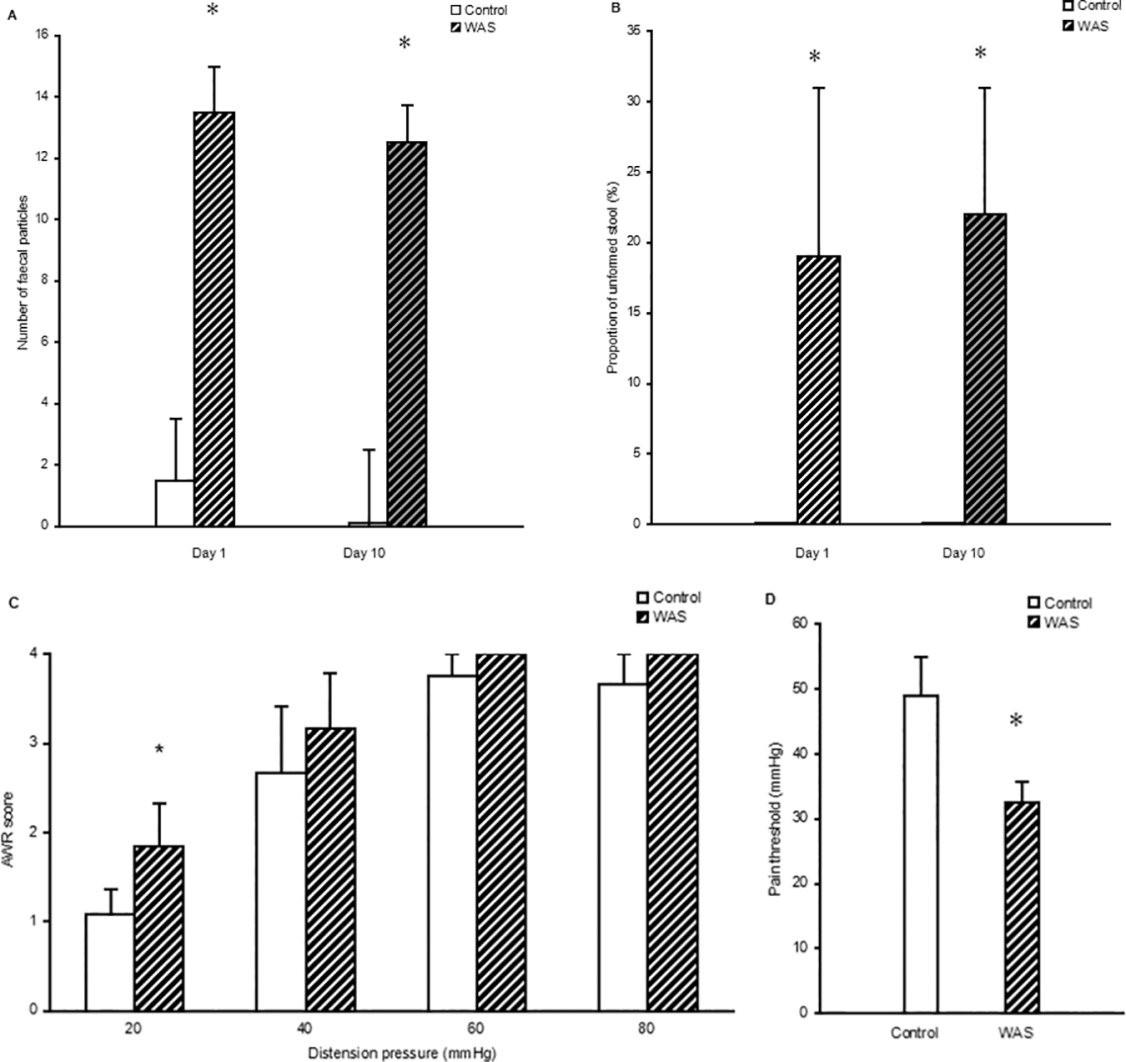
Water avoidance stress mimics diarrhea and visceral hypersensitivity. (A) Shown is the number of fecal particles excreted during 1 h on the day 1 and day 10 of water avoidance stress (WAS, n = 18) and control (n = 9). (B) Shown is the proportion of unformed stool excreted during 1 h on the day 1 and day 10 of WAS and control. (C) Shown is the abdominal withdrawal reflex (AWR) score in WAS group (n = 12) and control group (n = 6). (D) Shown is the pain threshold in WAS and control groups (independent sample *t* test, *differences between the WAS group and control group, *P* < 0.05).

On the first day of WAS modeling, the number of fecal particles excreted during 1 h increased significantly (13.5, IQR = 6 *vs* 1.5, IQR = 3.25, independent sample *t* test, *P* < 0.05), and the proportion of unformed stool was elevated significantly (19%, IQR = 17% *vs* 0%, IQR = 0, independent sample *t* test, *P* < 0.05) (Fig 1A). These significant increases in fecal particle number and proportion of unformed stool differences were long-lasting. Ten days later, the number of fecal particles and the proportion of unformed stool were still significantly higher than those in the control group (12.5, IQR = 3.75 *vs* 0, IQR = 2.5, independent sample *t* test, *P* < 0. 05; 22%, IQR = 23% *vs* 0%, IQR = 0, independent sample *t* test, *P* < 0.05) (Fig 1B). This result shows that the rats had developed obviously diarrhea.

After establishment of the animal model, VHS was measured in WAS rats and control rats. The AWR score in WAS group was significantly higher than that of control group at a distension pressure of 20 mmHg (1.84, IQR = 0.79 *vs* 1.09, IQR = 0.45, independent sample *t* test, *P* < 0.05). However, when the distension pressure was 40 mmHg to 80 mmHg, the AWR score was comparable between both groups (*P* > 0.05) (Fig 1C). Moreover, the pain threshold was reduced dramatically in the WAS group compared with control group (32.5, IQR = 3.75 *vs* 49, IQR = 13.5, independent sample *t* test, *P* < 0.05) (Fig 1D).

### IMO improved visceral hyperalgesia of WAS rats

**Fig 2:**
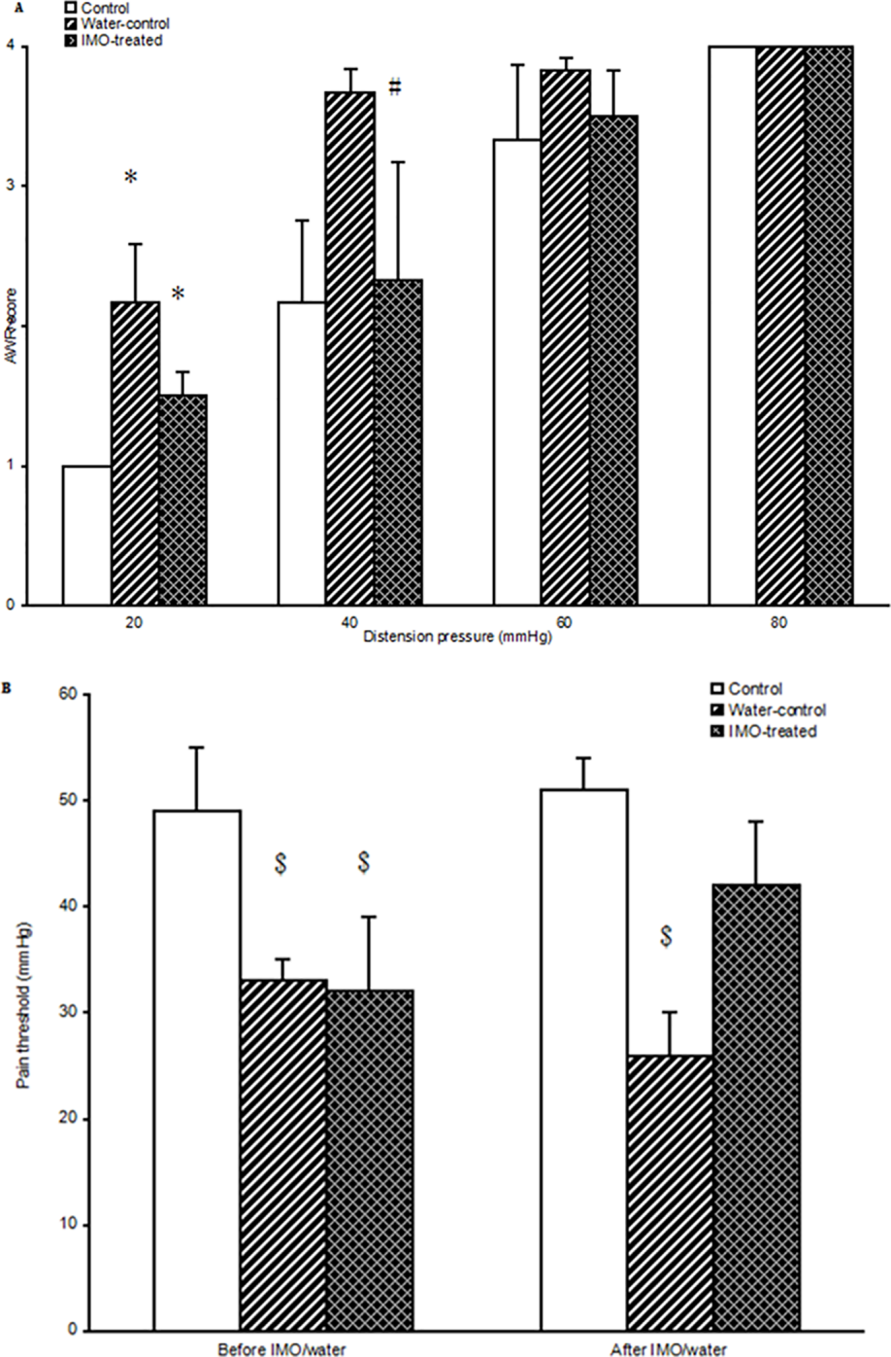
Isomalto-oligosaccharides (IMO) improved visceral hyperalgesia of water avoidance stress (WAS) rats. (A) Shown is the abdominal withdrawal reflex (AWR) score after inventions. (B) Shown is the pain threshold before and after the interventions. Median and interquartile (IQR). (n = 6 in control group, n = 5 in water-control group, and n = 7 in IMO-treated group. LSD test, differences between control group and water-control group, IMO-treated group and control group, *P < 0.05 *vs* control group; differences between IMO-treated group and water-comtrolled group, ^#^*P* <0.05; differences between water-control group, IMO-treated group and control group at the same period, ^$^ *P* < 0. 05).

After IMO treatment, the AWR score was similar to that of the water-control group when the distension pressure was 20 mmHg (1.5, IQR = 0.34 *vs* 2.17, IQR = 1.34, LSD test, *P* > 0.05). However, when the distension pressure was 40 mmHg, AWR score in IMO-treated group was significantly lower than that of water-control group (2.33, IQR = 1.17 *vs* 3.67, IQR = 0.67, LSD test, *P* < 0.05) and comparable to that of control group (2.33, IQR = 1.17 *vs* 2.17, IQR = 1.08, LSD test, *P* = 0.43). IMO treatment had no influence on AWR score when distension pressure was 60 or 80 mmHg (Fig 2A).

After 14 days of intervention, the pain threshold in water-control group was significantly lower than that of control group (26, IQR = 7, *vs* 51, IQR = 6, LSD test, *P* < 0.05). However, the pain threshold in IMO-treated group showed an increased trend and was comparable to that of control group (42, IQR = 23 *vs* 51, IQR = 6, LSD test, *P = 0*.*13*) (Fig 2B).

### IMO treatment had no significant influence on the intestinal transit rate

In IMO-treated group, the intestinal transit rate was 67.46%, IQR = 11.11%, which is comparable to that in water-control group (64.21%, IQR = 41.73%, LSD test, *P = 0*.*39*) and control group (54.62%, IQR = 26.17%, LSD test, *P = 0*.*18*).

### IMO did not influence the distribution of gut microbiota

After treatment, the number of bacterial colonies was determined using bacterial culture and one way analysis of variance was employed to compare the number of different bacteria (*Escherichia coli*, *Lactobacillus* and *Bacteroides*) among groups. The results showed that the number of bacterial colonies was comparable among the three groups (Table 1).

**Table 1:** Effect of IMO on the distribution of gut microbiota in WAS rats.

log10(CFU g^-1^)	Control(IQR)	water-control(IQR)	IMO-Treated(IQR)	*P* value
*E*.*coli*	4.49 (0.36)	4.29 (0.6)	4.20 (1.67)	0.72
*Lactobacillus*	6.43 (0.65)	6.21(0.32)	6.34 (0.46)	0.79
*Bacteroides*	5.19 (0.23)	5.56 (0.46)	5.09 (0.39)	0.58

WAS: Water avoidance stress, IMO: isomalto-oligosaccharides; n = 9 in each group. *P* value by Student-Newman-Keuls test.

### IMO repaired intestinal mucosal injury in the WAS rats

**Fig 3:**
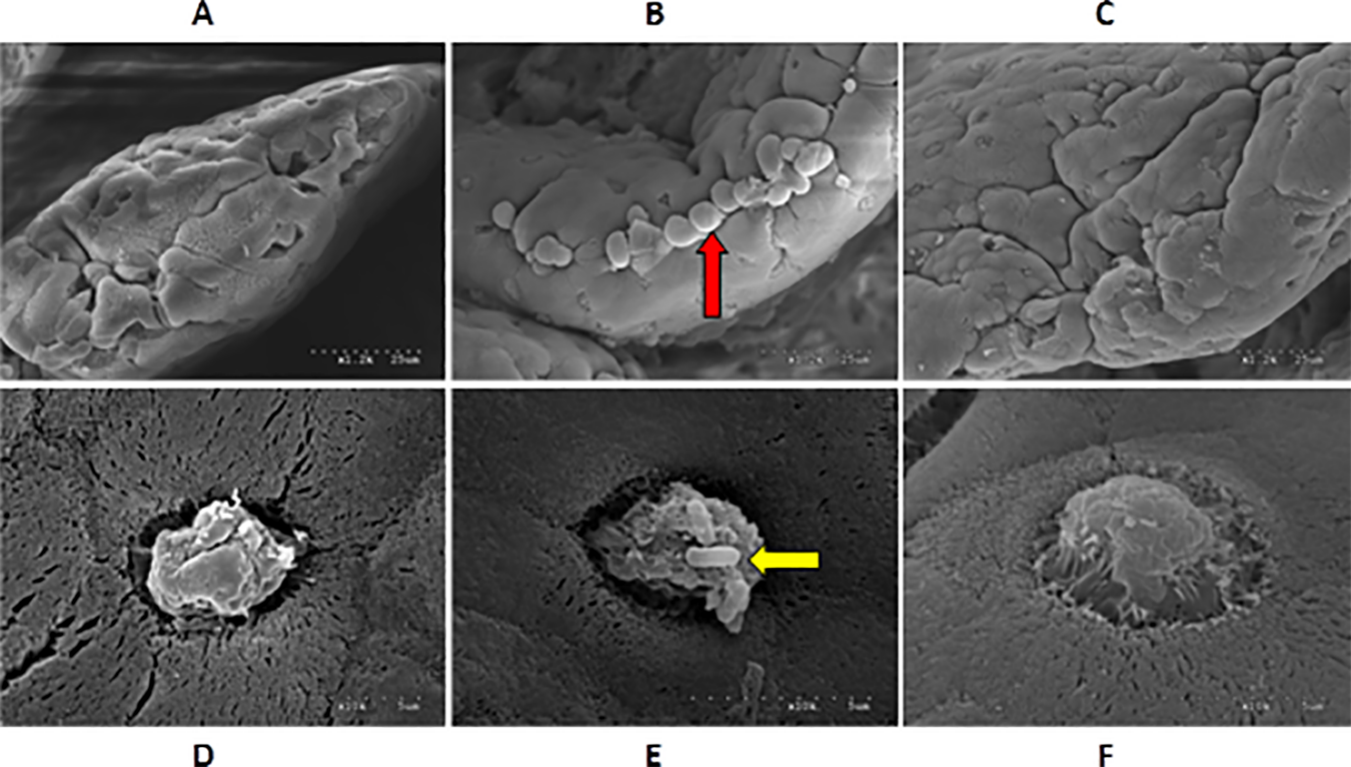
Ultrastructure of mucosal epithelial cells of ileum under a scanning electron microscope. (A) and (D): control group; (B) and (E): water-control group; (C) and (F): IMO-treated group. A, B, C: ×1200; D, E, F: ×10000. Long arrow: secretory granules at the opening of the mucosal glands; short arrow: bacilli on goblet cells.

The ileum tissue samples were collected from rats in different groups and subjected to H&E staining. Inflammatory changes were not seen under the microscope. SEM was performed to observe the ultrastructure of the intestinal epithelium of the ileum. When compared to control group (Fig 3A and 3D), many secretory granules were seen at the opening of mucosal glands in water-control group (Fig 3B and 3E), the regional microvilli were shed, goblet cells became pyknotic, and some bacilli were found on the cells (Fig 3E); The distance between epithelial cells increased, and the microvilli were smaller and showed an uneven distribution. After IMO treatment, secretory granules were no longer observed at the opening of mucosal glands, mild shedding of regional microvilli was seen (Fig 3C), the morphology of the goblet cells was similar to that of goblet cells in control group, and no bacilli were found on the cells. Furthermore, the distance between epithelial cells increased, the microvilli were smaller, and the distribution of microvilli was acceptably even (Fig 3F).

### WAS and IMO had no influence on serum cytokines

Serum concentrations of IL-10, IL-12 and TNF-a of the three groups are shown in Table 2 (LSD test, all *P* > 0.05).

**Table 2:** Serum concentration of cytokines in different groups.

Cytokines(pg mL^-1^)	Control(IQR)	water-control(IQR)	IMO-Treated(IQR)	*P* value
IL-10	258.00 (229.72)	393.33 (317.53)	402.00 (308.33)	0.68
IL-12	73.86 (33.27)	63.26 (36.00)	56.82 (33.04)	0.73
TNF-α	596.67 (555.55)	818.52 (847.91)	779.63 (685.86)	0.87

IMO: isomalto-oligosaccharides; n = 9 in each groups. *P* value by Student-Newman- Keuls test.

## Discussion

The findings of this study led us to conclude that IMO can improve WAS-induced VHS, and this improvement was mainly achieved by repairing stress injury on the ultrastructure of intestinal mucosal epithelial cells.

Available data indicated that VHS is a hallmark for IBS patients, and the disruption of the mucosal barrier and activation of regional inflammation play important roles in the development and maintenance of VHS[26–30]. But previous studies have not investigated whether abnormalities in the ultrastructure of the mucosal epithelium were present. Abnormalities in some receptors of the nervous system (such as the transient receptor potential vanilloid-1 receptor and the cannabinoid receptor) maybe involved in the pathogenesis of VHS[28]. Probiotic *Lactobacilli* may upregulate the expression of cannabinoid and opioid receptors in intestinal mucosal epithelial cells, inducing effects that are similar to morphine-induced anesthesia, and this upregulation may relieve VHS[13]. In addition, the protective effects of probiotics may be related to other mechanisms involved in intestinal permeability and pain transmission (such as reduction in the expression of occludin and junctional adhesion protein-A and prevention of an increase in intestinal permeability)[14,31,32]. Previous studies have also reported that probiotics were able to attenuate increased *Fos* expression in the spinal cord and supraspinal sites after stress[33]. Moreover, probiotics could also obstruct the increase of substance P level after intervention to stress or antibiotics use in order to inhibit VHS[12].

Few studies have been conducted to investigate the effect of prebiotics on IBS[34]. It is speculated that prebiotics may regulate the distribution of gut microbiota, increase fecal weight and frequency of defecation to improve the symptoms of constipation, and exert therapeutic effects. Non-absorbable sugar in the human body not only increases liquid volume in the colon, but also stimulates the gut microbiota, acidifies the right colon, and increases the bacterial enzymes that are able to metabolize carbohydrate (such as β-galactosidase)[35]. For healthy subjects, prebiotics of different types and/or at different doses may increase the amount of fecal *Bifidobacteria*[36,37]. However, the therapeutic effects of prebiotics on VHS are poorly understood.

In the present study, WAS was introduced to induce chronic stress in order to cause diarrhea and VHS in rats, which can mimic the clinical manifestations of IBS. In our study, after establishment of the rat model, the AWR score showed an increased response to colorectal distension at a lower pressure and a reduction in pain threshold, suggesting VHS. Two weeks after establishing the VHS model, these alterations were still apparent in the water-control rats. This suggests that the effect of WAS is sustained and measurable.

IMO is a non-fermentable prebiotic and can selectively increase the amount of lactobacillus in the gut of rats. Unlike the fructose widely used in the treatment of IBS with predominant constipation, IMO is unable to increase intestinal gas production and therefore will not worsen abdominal distension. A placebo-controlled, diet-controlled trial showed that treatment with a IMO-rich diet for 4–8 weeks in the elderly with constipation increased the frequency of spontaneous defecation, elevated the amount of fecal *Lactobacillus*, *Bifidobacterium* and *Enterobacteriaceae*, and decreased the amount of *Clostridium* and increased fecal wet weight by 24%[21]. In the present study, the results showed that VHS was improved after IMO treatment in WAS rats, an improvement characterized by reduction trend in AWR score responding to colorectal distension at a pressure of 20 mmHg, significant reduction in AWR score (close to that in control group) responding to colorectal distension at 40 mmHg, and an increase in pain threshold responding to colorectal distension. Our findings indicate that 2-week IMO treatment may improve VHS in IBS rats, but has no significant influence on intestinal motility.

This study aimed to explain the therapeutic effect of IMO on VHS in IBS rats from the aspects of change in gut microbiota, mucosal barrier function and systemic immune response. Xu et al.[32] found that the intestinal contents of IL-17, IL-6 and TNF-α increased significantly in WAS rats, and were reduced after rifaximin treatment, while the contents of IL-10, IFN-1β and IFN-γ remained unchanged before and after WAS intervention. In the present study, bacterial culture was performed in freshly collected stool, and the serum contents of IL-10, IL-12 and TNF-α were also determined. The results showed that the gut flora and systemic immune response remained unchanged after the improvement of VHS due to IMO treatment. This discrepancy might be ascribed to differences in detection methods. SEM showed that WAS caused damage to the ultrastructure of intestinal epithelium (such as a large amount of secretory granules at the opening of the glands, focal shedding of microvilli, shrinkage of goblet cells, aggregation of bacilli on goblet cells, increase in the distance between epithelial cells and uneven distribution of microvilli in ileum). Our study for the first time indicated that IMO could reverse damage to the ultrastructure of intestinal mucosal epithelium in WAS-induced IBS rats, an action that paralleled the improvement of VHS in these rats. We speculate that damages in the ultrastructure of intestinal mucosa play an important role in the induction and perpetuation of VHS, or else serve as a manifestation of VHS, or a trigger causing VHS. In the WAS-induced damages to the intestinal mucosal ultrastructure, the distance between epithelium increased, focal microvilli were shed, and microvilli showed an uneven distribution. These changes suggested mucosal edema. In addition, the presence of a large amount of secretory granules, shrinkage of goblet cells and aggregation of bacilli on goblet cells indicated abnormality in secretory function and an increase in bacterial adherence to the mucosa. However, these ultrasturctural changes were not observed under light microscopy. Thus, we postulate that stress-induced activation of systemic or local neuroimmunological reaction may cause damage to the ultrastructure of intestinal mucosa and subsequent induce VHS, and that a local inflammatory reaction in the intestinal mucosa in turn worsens the immune reaction, causing persistence of VHS. IMO-induced improvement for the damages of intestinal mucosal epithelium is the basis the VHS reversal seen in WAS-treated rats.

IMO is partly digestible and therefore the dose of IMO is critical for therapeutic efficacy. IMO at 10 g/d is required in human body to increase the amount of *Bifidobacterium* significantly[38]. That is 200 mg/d for a rat weighing 200 g. In the present study, 5% IMO (2 mL) was administered twice daily, a dose equivalent to the dose of 200 mg/d. However, IMO at this dose failed to stimulate the proliferation of gut flora. Comparing with other studies addressing IMO and microbiome in rats, we noted several differences between our study and piblications. First of all our dosage might be insufficient, as two experiment[39,40] indicated that IMO at a dose of 8g/kgwt per day was capable of microbiome regulation by increasing lactobacilli or decreasing *Clostridium*. Besides, the sensitivity of detection was limited using bacterial culture, this was a methological limitation of our study. Maybe 16S sequencing of bacterial DNA isolated from the collected fecal samples using the MiSeq platform could be more sensitive[41]. However, we did noticed a similiar report from Xiaoxi B. Lin et al[42]. In this study, they declared the reduction in irinotecan-induced (a chemotherapy medication) intestinal toxicity in rats by non-digestible carbohydrates (also including IMO) did not correlate to stimulation of specific bacterial taxa. They found that an increased cecal production of butyrate mediated symptom improvement. Back to our study, not by regulating the flora,. undigested IMO might exert protective effects on the intestinal mucosa that might be associated with a physical effect of IMO (such as an ultrainfiltration action and local covering of mucosa), and the resulting improvement of the ultrastructure of intestinal mucosa is helpful to block the vicious cycle of local inflammation at the VHS status.

## Summary

Taken together, IMO can significantly improve the visceral hyperalgesia of WAS-induced IBS rats and repair the damages on epithelial ultrastructure of intestinal mucosa. Our findings provide evidences for the therapeutic effect of prebiotics on IBS.

## Supporting information

S1 File This is the certification letter of language editing. (PDF)

S2 File This is the original data set of this experiment. (RAR)

## Abbreviations

AWR: abdominal withdrawal reflex
ELISA: enzyme-linked immunosorbent assay
IBS: irritable bowel syndrome
IMO: isomalto-oligosaccharides
ITR: intestinal transit rate
SEM: scanning electron microscopy
SPF: specific pathogen free
VHS: visceral hypersensitivity
WAS: water avoidance stress
