# Supplementary material for: Isomalto-oligosaccharides ameliorate visceral hyperalgesia with repair damage of ileal epithelial ultrastructure in rats
Source: PLoS One. 2017 Apr 24;12(4):e0175276. doi: 10.1371/journal.pone.0175276 (PMC5402968; doi:10.1371/journal.pone.0175276)
Supplement: S1 File — (PDF) [file pone.0175276.s001.pdf]

ZTC011014
Date of Issue: Feb. 19, 2014

CERTIFICATION

This is to certify that a medical editor who is a native English speaker associated with MedCom Asia, Inc., has edited the manuscript entitled "**Isomalto-oligosaccharides improve rat visceral hypersensitivity to acute stress by repairing intestinal epithelial ultrastructure**".

This manuscript has been sent back to the author on Feb. 19, 2014. If there is any change on the manuscript by the author after the above mentioned date, this certificate is invalid.

The medical editor who edited the manuscript has been a professional editor and writer for several years and is a member of American Medical Writers Association.

MedCom Asia, Inc.

2 F, No. 60, Minquan Rd., Xindian City
Taipei County 231, Taiwan
Tel: +886-2-2912-6638; Fax: +886-2-2912-6648
Email: service@medcom.com.tw
Web: http://www.asiamedcom.com/eng/
